# Sexual minority status and symptoms of psychosis: The role of bullying, discrimination, social support, and drug use – Findings from the Adult Psychiatric Morbidity Survey 2007

**DOI:** 10.1111/papt.12242

**Published:** 2019-07-25

**Authors:** Robert Qi, Jasper Palmier‐Claus, Juliette Simpson, Filippo Varese, Richard Bentall

**Affiliations:** ^1^ Institute of Health and Life Sciences University of Liverpool UK; ^2^ Spectrum Centre for Mental Health Research Lancaster University UK; ^3^ Lancashire Care NHS Foundation Trust UK; ^4^ Division of Psychology and Mental Health University of Manchester UK; ^5^ Greater Manchester Mental Health NHS Foundation Trust UK; ^6^ Department of Psychology University of Sheffield UK

**Keywords:** adversity, hallucinations, paranoia, psychosis, sexual minorities

## Abstract

**Objective:**

Sexual minorities have an increased risk of psychosis, potentially explained by experiences of social adversity. Sexual minorities may also have a specific risk of paranoid symptoms. The current study aimed to determine whether sexual minorities have increased risk of psychosis, whether they have a specific increased risk of paranoia when compared to auditory verbal hallucinations (AVHs), and whether social adversity such as bullying, recent discrimination, lack of social support, and drug use can explain this risk.

**Methods:**

The study used data from the Adult Psychiatric Morbidity Survey 2007 (*n* = 7,403), exploring both sexual identity and past sexual behaviour. Associations between sexual minority status and probable psychosis, paranoia, and AVH were analysed using logistic regression. Mediation analysis was also conducted using the Karlson–Holm–Breen method, with bullying, recent discrimination, social support, and drug use as mediators assessing pathways between sexual minority status and paranoia/AVH. Socio‐demographic confounders were included in analyses.

**Results:**

Sexual minority status did not significantly predict probable psychosis. Findings generally indicated a specific association between sexual minority status and paranoia when contrasted with AVH. However, sexual behaviour remained significantly associated with AVH in logistic regression models. Bullying, lack of social support, and drug use partially mediated the association between sexual minority status and paranoia.

**Conclusions:**

Sexual minority status appears to have a specific association with paranoia symptoms, which may be partially explained by experiences of social adversity. However, the cross‐sectional nature of the study limits direct inference about causality of such symptoms.

**Practitioner points:**

- Sexual minority groups may be more likely to experience symptoms of paranoia.
- It may be important to consider experiences of social adversity such as bullying, lack of social support, and also history of drug use in the context of paranoia within these groups.

## Background

Sexual minorities (non‐heterosexual groups) may be at greater risk of poor mental health when compared to heterosexuals, showing higher prevalence of depression, anxiety, suicidal behaviour, drug abuse, and severe mental illness (Kidd, Howison, Pilling, Ross, & McKenzie, 2016; King *et al*., 2008). Large nationally representative surveys in the United Kingdom and the United States have shown an increased risk of psychotic disorders in sexual minorities (Bolton & Sareen, 2011; Chakraborty, McManus, Brugha, Bebbington, & King, 2011). Further research is needed to better understand this association in order to reduce the increased risk of distressing psychotic experiences within sexual minority populations.

The reasons for the association between sexual minority status and psychosis are likely multifaceted, but may be strongly related to social adversity and disadvantage (Heinz, Deserno, & Reininghaus, 2013; Wicks, Hjern, Gunnell, Lewis, & Dalman, 2005). Sexual minorities face higher levels of bullying and discrimination as a result of their sexual orientation (King *et al*., 2011) and experience reduced social support, which has been linked to worse mental health outcomes (Lehavot & Simoni, 2007; Williams, Connolly, Pepler, & Craig, 2005). Indeed, isolation may predate the onset of symptoms (Gayer‐Anderson & Morgan, 2013) and prevent recovery (Pruessner, Iyer, Faridi, Joober, & Malla, 2011). Using a Dutch nationally representative survey, Gevonden *et al*. (2013) found that adversity such as bullying, discrimination, and drug use partially mediated the relationship between sexuality (based on avowed sexual identity and reported actual sexual contact with others of the same sex) and an increased risk of psychotic symptoms. Investigation of mediators between minority status and psychosis may help to identify important targets for prevention and intervention.

Due to the broad diagnostic categories that define clinical disorders, the term psychosis may encompass heterogeneous phenomena (Bentall, 2004). Assessing diagnostic groups may present a hindrance to gaining a deeper understanding of the factors causing psychosis, and some researchers suggest using narrower symptom dimensions (van Os & Kapur, 2009) to investigate and treat specific symptoms (Bentall & Fernyhough, 2008). It is plausible that minority group status may also have symptom‐specific effects. Indeed, some ethnic minority groups have been found to have a particularly increased risk of paranoia amongst first‐episode psychosis patients (Veling, Selten, Mackenbach, & Hoek, 2007). Similarly, sexual minority status may also be specifically associated with paranoia symptoms.

Social adversity faced by sexual minorities may lead to paranoia in a number of ways. Psychological studies have shown that paranoia may stem from the over‐anticipation of threat from the environment (Freeman, Garety, Kuipers, Fowler, & Bebbington, 2002), which could be exacerbated by social adversity (Green & Phillips, 2004). Consistent with this account, data from a prospective study indicated that perceived discrimination in minority groups predicted paranoid delusions, but not hallucinatory experiences (Janssen *et al*., 2003). It has also been argued that negative social identity can explain the elevated levels of paranoia in disadvantaged groups such as ethnic minorities (McIntyre, Elahi, & Bentall, 2016). A positive social identity is related to higher self‐esteem (Branscombe, Schmitt, & Harvey, 1999), while low self‐esteem predicts paranoid delusions (Freeman *et al*., 1998) and persistence of long‐term paranoia (Fowler *et al*., 2012). This combination of increased threat anticipation and poor social identity/self‐esteem might be expected to specifically increase the risk of paranoid symptoms.

In contrast, there are reasons for expecting that auditory verbal hallucinations (AVH) will be less likely to be associated with sexual minority status. Though AVH and paranoia symptoms commonly occur together (van Os, Linscott, Myin‐Germeys, Delespaul, & Krabbendam, 2009), they are thought to be the product of distinct psychological processes, specifically source monitoring deficits and dissociative mechanisms (Brookwell, Bentall, & Varese, 2013; Pilton, Varese, Berry, & Bucci, 2015). It is thought that risk factors for AVH are associated with specific adversities such as childhood sexual abuse (Bentall *et al*., 2014). The finding that perceived discrimination in minority groups predicts paranoid delusions but not hallucinations (Janssen *et al*., 2003) is consistent with the hypothesis that sexual minority status will have little impact on risk of AVH.

Using the Adult Psychiatric Morbidity Survey 2007 (APMS 2007; McManus, Meltzer, Brugha, Bebbington, & Jenkins, 2009), the current study assessed whether sexual minority status was related to probable psychosis, and also whether there were specific effects on symptoms of paranoia and auditory verbal hallucinations. We also explored the contribution of four potential mediators (bullying, recent discrimination due to one's sexuality, drug use, and social support) on these symptoms. Our primary hypothesis was that sexual minority status (both avowed identity and reported sexual behaviour) would significantly predict probable psychosis. Our second hypothesis was that sexual minority status would significantly predict paranoia, but not AVH, after controlling for symptom co‐occurrence. Our final hypothesis was that the four mediator variables would partially mediate any relationship between sexual minority status and paranoia.

## Method

### Sample

The APMS 2007 was carried out between October 2006 and December 2007 and employed a multistage stratified probability sampling design. Individuals aged 16 years and above living in private households were identified for interview in England using postcodes. From 13,171 eligible households, 7,403 individuals completed the first phase (although a second phase involved clinical interviews with a subsample, these data are not used in this study, with the exception that they contributed to the definition of probable psychosis; see below). Researchers administered computer‐assisted interviews and self‐completed questionnaires using laptops to obtain data on topics including physical health, mental health, service use, religion, social capital, discrimination, and experiences of childhood trauma. For more information, see the full report (McManus *et al*., 2009).

### Measures

#### Probable psychosis

The APMS 2007 screened for psychosis experiences during phase one using the Psychosis Screening Questionnaire (PSQ; Bebbington & Nayani, 1995) and then interviewed a subsample of participants with the Schedule for Clinical Assessment in Neuropsychiatry (SCAN; Wing *et al*., 1990) in phase two. Of the 7,403 respondents who completed a phase one interview, 313 (4%) met at least one of the psychosis screening criteria and therefore were eligible for a phase two interview. Of those eligible, 190 (61%) completed a productive phase two interview.

Probable psychosis is a binary variable derived from phase one and two data, using the following procedure:

1. For those who screened positive for psychosis at phase one and had a SCAN assessment, the results of the SCAN were used.
2. For those who screened negative for psychosis at phase one, it was assumed that these were true negatives regardless of whether or not a SCAN assessment was completed.
3. For those who screened positive for psychosis at phase one but did not have a SCAN assessment (e.g., due to refusal or non‐contact), those meeting one psychosis screen criterion were assigned a negative outcome, while those meeting two or more criteria were assigned a positive outcome.

The ‘Probable Psychosis’ variable represents a slightly less conservative method for estimating psychosis prevalence when taking into account the non‐response to phase two SCAN assessments, and allows for consistent comparison across APMSs.

#### Paranoia and auditory verbal hallucinations

The PSQ (Bebbington & Nayani, 1995) has five sections to identify psychotic‐like experiences that may have occurred within the past year: mania/hypomania, thought control, paranoia, strange experiences, and auditory verbal hallucinations (AVH). Each section has an initial question, followed up by one or two questions to determine severity. The paranoia and AVH sections of the PSQ were of interest in the present study and were scored as binary variables, with the highest severity being scored as 1, and all others scored as 0.

The highest severity of paranoia was identified if respondents answered yes to the question, ‘Have there been times that you felt that a group of people was plotting to cause you serious harm or injury?’ This represents a narrow definition of paranoia, encompassing thoughts of deliberate acts of harm and plotting against an individual as has been used in previous publications using this data set (Bentall, Wickham, Shevlin, & Varese, 2012; Wickham, Taylor, Shevlin, & Bentall, 2014). The highest severity of AVH was identified if respondents answered yes to the question, ‘Did you at any time hear voices saying quite a few words or sentences when there was no one around that might account for it?’

Questions from the Sexual Orientation and Partnership section were used to generate two variables regarding sexuality based on sexual identity and sexual behaviour, using a similar method which has been described in a previous study (Chakraborty *et al*., 2011). Analysis will be conducted using both variables separately.

#### Sexual identity

Respondents were asked one of two possible questions regarding sexual identity and were randomly allocated to one of the questions. The first was ‘Which statement best describes your sexual orientation? This means sexual feelings, whether or not you have had any sexual partners’ where the possible responses include ‘Entirely heterosexual (attracted to persons of the opposite of sex)’, ‘Mostly heterosexual, some homosexual feelings’, ‘Bisexual (equally attracted to men and women)’, ‘Mostly homosexual, some heterosexual feelings’, ‘Entirely homosexual (attracted to persons of the same sex)’, and ‘Other’. The second question was ‘Please choose the answer below that best describes how you think of yourself…’ where the possible responses include ‘completely heterosexual’, ‘mainly heterosexual’, ‘bisexual’, ‘mainly gay or lesbian’, ‘completely gay or lesbian’, or ‘Other’.

A response of either ‘Entirely heterosexual (attracted to persons of the opposite of sex)’ to the first question or ‘completely heterosexual’ to the second question was counted as ‘Heterosexual’ with all other responses being counted as ‘Non‐Heterosexual’.

#### Sexual behaviour

Respondents were asked one of two questions regarding the sex of their past sexual partners and were randomly allocated to one of the questions. The first question is limited to sexual intercourse while the second relates to sexual contact, representing a broader definition. The first question was ‘Have your sexual partners been…’ with possible responses including ‘only opposite sex’, ‘mainly opposite sex but some same sex partners’, ‘mainly same sex but some opposite sex partners’, ‘only same sex’, or ‘I have not had a sexual partner’. The second question was ‘Sexual experience is any kind of contact with another person that you felt was sexual (it could be just kissing or touching, or intercourse, or any other form of sex). Has your sexual experience been…’ where the possible responses included ‘Only with (women/men) or a (woman/man), never with a (man/woman)’, ‘More often with (women/men), and at least once with a (man/woman)’, ‘About equally often with (women/men) and (men/women)’, ‘More often with (men/women), and at least once with a (woman/man)’, ‘Only with (men/women) or a (man/woman), never with a (woman/man)’, or ‘I have never had any sexual experience with anyone at all’.

A response of either ‘only opposite sex’ to the first question or ‘Only with (women/men) or a (woman/man), never with a (man/woman)’ was counted as ‘Heterosexual’. Any other response was counted as ‘Non‐Heterosexual’, with the exception of the answers ‘I have not had a sexual partner’ and ‘I have never had any sexual experience with anyone at all’ which were excluded. As a result, this variable excludes the sexually inactive population.

#### Bullying

A binary variable for bullying during any point in the lifetime from the Stressful Life Events section. Respondents had to select ‘bullying’ from a list of options on a card following the question, ‘Now looking at this card, could you tell me if you have ever experienced any of these problems or events, at any time in your life?’ along with ‘Within last 6 months’, ‘more than 6 months ago, but since the age of 16’, or ‘more than 6 months ago, and before the age of 16’ in response to the question ‘When did that happen?’

#### Discrimination (sexuality)

One question from the Discrimination section was used to generate a binary mediating variable for discrimination. The question used was ‘Have you been unfairly treated in the last 12 months, that is since (date), because of your sexual orientation?’ with a response of ‘No’ equating to 0 and ‘Yes’ equating to 1.

#### Social support

Seven items were used to identify the level of social support each respondent felt that they had from family and friends. Participants were asked to respond ‘not true’, ‘partly true’, or ‘certainly true’ to a series of statements, including whether family and friends did things to make them happy, made them feel loved, could be relied on no matter what, would see that they were taken care of no matter what, accepted them just the way they are, made them feel an important part of their lives, and gave them support and encouragement. Each respondent could have a score between 0 and 14, creating a 15‐point scale.

#### Drug use

Any drug use within the last year was added as a binary mediating variable (1 = Yes, 0 = No). The drugs included were as follows: cannabis, amphetamines, cocaine and crack cocaine, ecstasy, heroin, LSD, magic mushrooms, methadone, tranquilizers, amyl nitrate, anabolic steroids, and glues/solvents.

#### Covariates

A number of covariates were included in the analyses. These included age and sex. Ethnicity was also added as a categorical variable where 0 equated to white (British and non‐British) and 1 equated to all others. Finally, the highest level of educational qualification attained by respondents was included, consisting of six categories (degree, teaching/HND/nursing, A‐levels, GCSE/equivalent, foreign/other, and no qualification).

### Statistical analysis

The survey data were weighted to represent the national population, taking account of non‐response, size of household, characteristics of non‐responding households, and the profile of age and gender within the government office region.

All analyses were carried out in Stata 13.1 for Windows. The ‘survey’ commands in Stata were used, which allow for the use of clustered data modified by probability weights. All analyses were conducted on complete data, where respondents who had any missing data for relevant variables were excluded. First, logistic regressions were used to assess the relationship between the independent variable for sexuality (using sexual identity and sexual behaviour variables separately), and probable psychosis, paranoia, and AVH. Each logistic regression model was estimated without adjustment (Model 1) and when adjusting for all relevant covariates including age, gender, ethnicity, and highest educational level (Model 2). In Model 2, as in previous publications using this data set (Bentall *et al*., 2012; Wickham *et al*., 2014), AVH was included as a covariate when analysing paranoia, and paranoia was used as a covariate when analysing AVH, to control for co‐occurrence of symptoms.

**Figure 1:**
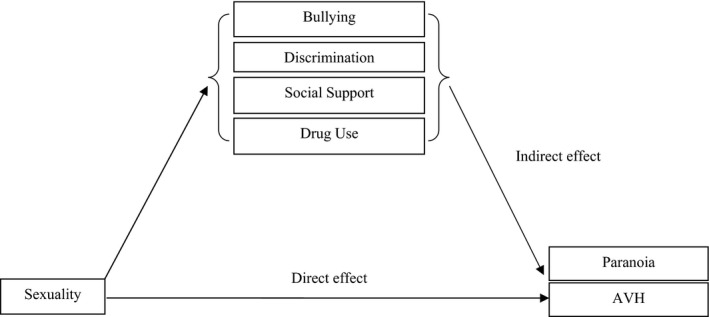
Illustration of mediation model used in the analysis. The mediator variables bullying, discrimination, social support, and drug use were allowed to co‐vary. Covariates included sex, age, ethnicity, highest educational level, and paranoia/AVH. The total effect of sexuality (using sexual identity and sexual behaviour separately) on paranoia and AVH individually was decomposed into direct and indirect effects. The relative contribution of each individual mediator is estimated.

The role of bullying, discrimination (sexuality), social support, and drug use as mediators of the relationship between sexuality and paranoia/AVH symptoms were assessed using the Karlson–Holm–Breen (‐khb‐) command in Stata (Figure 1). This method of mediation analysis decomposes the total effect of a variable into direct and indirect effects (Breen, Karlson, & Holm, 2013) and can be used in logit models. Again, the independent variables for sexuality were used in separate analyses. In these analyses, the confounding effects of covariates on the decomposition were controlled for. These covariates comprised sex, age, ethnicity, highest educational level, and either paranoia or AVH. Confidence limits were derived using the delta method of Sobel (Sobel, 1982).

## Results

Table 1 shows the baseline characteristics of the sample stratified by sexuality, based on sexual identity and sexual behaviour separately. In terms of sexual identity, 6,811 (93%) respondents were heterosexual, while 502 (7%) were non‐heterosexual. In terms of sexual behaviour, 6,794 (95%) were heterosexual, while 352 (5%) were non‐heterosexual. Using both definitions of sexuality, groups differed significantly on all variables of interest apart from sex.

**Table 1:** Bivariate associations between probable psychosis, paranoia, auditory verbal hallucination, and sexual identity/behaviour

	Sexual identity				Sexual behaviour			
	Heterosexual (*n *=* *6,811) (%)	Non‐heterosexual (*n *=* *502) (%)	Missing (*n *=* *90) (%)	*p*‐Value	Heterosexual (*n *=* *6,794) (%)	Non‐heterosexual (*n *=* *352) (%)	Missing (*n *=* *257) (%)	*p*‐Value
Probable psychosis								
Yes	32 (<1)	6 (1)	2 (2)	.009	33 (1)	2 (1)	5 (2)	.007
No	6,779 (>99)	496 (99)	98 (98)		6,761 (99)	350 (99)	252 (98)	
Paranoia								
Yes	100 (1)	23 (5)	2 (2)	<.001	101 (1)	17 (5)	7 (3)	<.001
No	6,609 (97)	466 (93)	81 (90)		6,590 (97)	325 (92)	241 (94)	
–	102 (2)	13 (2)	7 (8)		103 (2)	10 (3)	9 (3)	
AVH								
Yes	58 (<1)	8 (2)	2 (2)	<.001	57 (<1)	8 (2)	3 (1)	<.001
No	6,722 (99)	491 (98)	84 (93)		6,710 (99)	342 (97)	245 (95)	
–	31 (<1)	3 (<1)	4 (5)		27 (<1)	2 (1)	9 (4)	
Age group								
16–34	1,430 (21)	159 (32)	14 (15)	<.001	1,408 (21)	124 (35)	71 (28)	<.001
35–54	2,336 (34)	190 (38)	17 (19)		2,354 (35)	140 (40)	49 (19)	
55–74	2,173 (32)	109 (22)	25 (28)		2,176 (32)	65 (19)	66 (25)	
>74	872 (13)	44 (8)	34 (38)		856 (12)	23 (6)	71 (28)	
Sex								
Male	2,944 (43)	219 (44)	34 (38)	0.572	2,932 (43)	153 (44)	112 (44)	0.985
Female	3,867 (57)	283 (56)	56 (62)		3,862 (57)	199 (56)	145 (56)	
Ethnicity								
White	6,312 (93)	443 (88)	52 (58)	<.001	6,288 (93)	327 (93)	192 (75)	<.001
Non‐White	479 (7)	56 (11)	11 (12)		488 (7)	23 (7)	35 (13)	
–	20 (<1)	3 (1)	27 (30)		18 (<1)	2 (<1)	30 (12)	
Educational level								
Degree	1,254 (18)	115 (23)	5 (6)	<.001	1,242 (18)	104 (30)	29 (11)	<.001
Teaching	504 (7)	37 (7)	1 (1)		514 (7)	22 (6)	6 (2)	
A‐level	871 (13)	63 (13)	4 (8)		867 (13)	51 (15)	20 (8)	
GCSE	1,692 (25)	117 (23)	8 (9)		1,683 (25)	84 (24)	49 (19)	
Foreign	260 (4)	24 (5)	2 (2)		267 (4)	12 (3)	7 (3)	
None	2,102 (31)	136 (27)	40 (44)		2,098 (31)	68 (19)	112 (44)	
–	128 (2)	10 (2)	30 (33)		123 (2)	10 (3)	35 (13)	
Social support								
0–7	209 (3)	27 (5)	4 (4)	<.001	212 (3)	13 (4)	15 (6)	<.001
8–14	6,563 (96)	467 (93)	53 (59)		6,541 (96)	336 (96)	206 (80)	
–	39 (1)	8 (2)	33 (37)		41 (1)	3 (<1)	36 (14)	
Experienced bullying								
Yes	1,231 (18)	157 (31)	4 (4)	<.001	1,207 (18)	142 (40)	43 (17)	<.001
No	5,580 (82)	345 (69)	86 (96)		5,587 (82)	210 (60)	214 (83)	
Discrimination (sexuality)								
Yes	6 (<0.1)	27 (5)	0 (0)	<.001	7 (<1)	24 (7)	2 (1)	<.001
No	6,767 (99)	473 (94)	33 (37)		6,751 (99)	326 (93)	196 (76)	
–	38 (<1)	2 (1)	57 (63)		36 (<1)	2 (<1)	59 (23)	
Drug use								
Yes	447 (7)	87 (17)	0 (0)	<.001	457 (7)	75 (21)	2 (1)	<.001
No	6,354 (93)	414 (83)	54 (60)		6,329 (93)	276 (78)	217 (84)	
–	10 (<1)	1 (<0)	36 (40)		8 (<1)	1 (1)	38 (15)	

### Relationship between sexual identity and probable psychosis, paranoia, and AVH

A summary of logistic regression analyses conducted using sexual identity is presented in Table 2. Sexual identity significantly predicted probable psychosis in Model 1 before adjusting for covariates, with non‐heterosexuals having higher levels of psychosis when compared with heterosexuals (OR 2.87, 95% CI 1.09–7.52). However, after adjusting for covariates in Model 2, the relationship between sexual identity and probable psychosis was no longer significant (OR 2.64, 95% CI 0.96–7.22), although there was a trend. Of the covariates, only age had a significant effect.

**Table 2:** Odds ratios and 95% CI for the effects of sexual identity on probable psychosis, paranoia, and AVH

	Model 1^a^		Model 2^b^	
	OR	*p*	OR	*p*
Probable psychosis				
Sexual identity	2.87 (1.09–7.53)	.033	2.64 (0.96–7.22)	.060
Sex			1.48 (0.69–3.14)	.311
Age			0.98 (0.97–1.00)	.021
Ethnicity			0.73 (0.21–2.63)	.635
Education			1.26 (0.96–1.65)	.091
Paranoia				
Sexual identity	3.07 (1.84–5.13)	<.001	2.30 (1.31–4.02)	.004
AVH			20.37 (9.26–44.81)	<.001
Sex			0.67 (0.44–1.04)	.073
Age			0.97 (0.96–0.98)	<.001
Ethnicity			2.93 (1.72–5.01)	<.001
Education			1.16 (1.01–1.33)	.041
AVH				
Sexual identity	2.65 (1.13–6.18)	.024	1.79 (0.75–4.28)	.190
Paranoia			19.31 (8.58–43.49)	<.001
Sex			1.74 (1.01–3.01)	.045
Age			0.98 (0.97–1.00)	.076
Ethnicity			0.51 (0.16–1.62)	.250
Education			1.19 (1.01–1.40)	.041

Notes

a Unadjusted.

b Adjusted for covariates.

Sexual identity significantly predicted both paranoia (OR 3.07, 95% CI 1.84–5.13) and AVH (OR 2.65, 95% CI 1.13–6.18) before adjusting for covariates in Model 1, with non‐heterosexuals having higher odds. After controlling for the co‐occurrence of AVH and other covariates, sexual identity remained a significant predictor of paranoia in Model 2 (OR 2.30, 95% CI 1.31–4.02). Of the covariates, age and education had significant effects on paranoia. Ethnicity also significantly predicted paranoia in this model, with non‐whites having significantly higher odds of paranoia compared to whites.

After controlling for the co‐occurrence of paranoia and other covariates, sexual identity no longer significantly predicted AVH in Model 2 (OR 1.79, 95% CI 0.75–4.28). In this model, sex significantly predicted AVH, with females having higher odds of AVH than males. There was also a significant effect of education.

### Relationship between sexual behaviour and probable psychosis, paranoia, and AVH

A summary of logistic regression analyses conducted using sexual behaviour is presented in Table 3. Sexual behaviour did not significantly predict probable psychosis before adjusting for covariates in Model 1 (OR 1.55, 95% CI 0.30–8.07), or after adjustment in Model 2 (OR 1.41, 95% CI 0.25–7.82). In the adjusted model, only age significantly predicted probable psychosis.

**Table 3:** Odds ratios and 95% CI for the effects of sexual behaviour on probable psychosis, paranoia, and AVH

	Model 1^a^		Model 2^b^	
	OR	*p*	OR	*p*
Probable psychosis				
Sexual behaviour	1.55 (0.30–8.07)	.602	1.41 (0.25–7.82)	.697
Sex			1.59 (0.72–3.52)	.252
Age			0.98 (0.97–0.99)	.001
Ethnicity			0.81 (0.23–2.88)	.748
Education			1.29 (0.96–1.73)	.088
Paranoia				
Sexual behaviour	2.67 (1.47–4.85)	.001	2.13 (1.17–3.87)	.013
AVH			19.98 (9.07–44.03)	<.001
Sex			0.71 (0.45–1.10)	.124
Age			0.96 (0.95–0.98)	<.001
Ethnicity			3.40 (2.02–5.74)	<.001
Education			1.18 (1.02–1.35)	.025
AVH				
Sexual behaviour	2.65 (1.13–6.18)	.024	2.47 (1.04–5.88)	.040
Paranoia			19.05 (8.49–42.72)	<.001
Sex			1.68 (0.98–2.89)	.059
Age			0.98 (0.97–1.00)	.069
Ethnicity			0.52 (0.16–1.70)	.278
Education			1.20 (1.02–1.42)	.029

Notes

a Unadjusted.

b Adjusted for covariates.

Sexual behaviour significantly predicted paranoia before adjusting for covariates, with non‐heterosexuals having higher odds of paranoia (OR 2.67, 95% CI 1.47–4.85). This relationship remained significant after adjusting for covariates and the co‐occurrence of AVH in Model 2 (OR 2.13, 95% CI 1.17–3.87). Of the covariates, age and education significantly predicted paranoia in this model. Ethnicity also significantly predicted paranoia, with non‐white participants having significantly higher odds of paranoia compared to whites.

Sexual behaviour significantly predicted AVH in Model 1 (OR 2.65, 95% CI 1.13–6.18), with non‐heterosexuals having higher odds of AVH. After controlling for covariates and the co‐occurrence of paranoia, non‐heterosexuals still had significantly higher odds of AVH (OR 2.47, 95% CI 1.04–5.88). Again, there was a significant effect of education on AVH in Model 2.

### Mediation analysis

Detailed results of the mediation analyses are presented in Table 4. Relative contribution of the individual mediators to the total effect and grand indirect effect is estimated as percentages.

**Table 4:** Mediation of the link between sexual identity/sexual behaviour and paranoia/AVH by bullying, discrimination (sexuality), social support, and drug use

Effect	Sexual identity (*n *=* *6,959)					Sexual behaviour (*n *=* *6,806)				
	OR	Robust *SE*	*z*	*p* > *z*	95% CI	OR	Robust *SE*	*z*	*p* > *z*	95% CI
Bullying, discrimination (sexuality), social support, and drug use as mediators of the effect of sexual identity/behaviour on paranoia^a^										
Total	2.38	0.69	2.99	.003	1.35–4.20	2.20	0.71	2.44	.015	1.17–4.15
Direct	1.89	0.58	2.07	.039	1.03–3.46	1.63	0.56	1.42	.155	0.83–3.20
Indirect	1.26	0.15	1.97	.049	1.00–1.58	1.35	0.19	2.20	.028	1.03–1.77
Bullying, discrimination (sexuality), social support, and drug use as mediators of the effect of sexual identity/behaviour on AVH^b^										
Total	1.45	0.81	0.66	.508	0.48–4.36	2.04	1.19	1.22	.221	0.65–6.42
Direct	1.12	0.67	0.19	.846	0.35–3.65	1.57	1.01	0.70	.483	0.44–5.57
Indirect	1.29	0.16	2.11	.035	1.02–1.63	1.30	0.19	1.78	.074	0.97–1.73

Notes

Data were weighted and controlled for the co‐occurrence of symptoms, sex, age, ethnicity, and educational qualification.

a Significant total, direct, and indirect effects found between sexual identity and paranoia. 26.4% of the total effect was mediated, 14.4% by bullying, 8.3% by drug use, and 5.3% by social support. Discrimination (sexuality) had a negative effect (−1.4%) on the link between sexual identity and paranoia. A significant total and indirect effect between sexual behaviour and paranoia was found. 38.12% of the total effect was mediated, 23.3% by bullying, 12.75% by drug use, and 3.52% by social support. Discrimination (sexuality) had a negative effect (−1.4%) on the link between sexual orientation (sexual partners) and paranoid delusions.

b Only a significant indirect effect between sexual identity and AVH was found. 43.6% of the effect was mediated by bullying, 37.5% by discrimination (sexuality), and 26.9% by social support. Drug use had a negative effect (−7.93%) on the link between sexual identity and auditory verbal hallucinations. No significant effects were found between sexual behaviour and AVH.

### Sexual identity and paranoia/AVH, with bullying, discrimination (sexuality), social support, and drug use as mediators

Total (OR 2.38, 95% CI 1.35–4.20), direct (OR 1.89, 95% CI 1.03–3.46), and indirect effects (OR 1.26, 95% CI 1.00–1.58) were significant for sexual identity and paranoia symptoms. 26.4% of the total effect was mediated, 14.4% by bullying, 8.3% by drug use, and 5.3% by social support. Discrimination (sexuality) had a negative effect (−1.4%). Only a significant indirect effect was found between sexual identity and AVH (OR 1.29, 95% CI 1.02–1.63). 43.6% of this effect was mediated by bullying, 37.5% by discrimination (sexuality), and 26.9% by social support, while drug use had a negative effect (−7.93%) on the link between sexual identity and AVH.

### Sexual behaviour and paranoia/AVH with bullying, discrimination (sexuality), social support, and drug use as mediators

A significant total (OR 2.20, 95% CI 1.17–4.15) and indirect effect (OR 1.35, 95% CI 1.03–1.77) was found regarding sexual behaviour and paranoia. 38.12% of the total effect was mediated, 23.3% by bullying, 12.75% by drug use, and 3.52% by social support. Discrimination (sexuality) had a negative effect (−1.4%). No significant effects were found between sexual behaviour and AVH.

## Discussion

The current study assessed whether sexual minority status (identity and behaviour) related to probable psychosis and whether there were specific effects on paranoia and AVH. Additionally, the authors explored whether bullying, drug use, recent discrimination due to one's sexuality, and social support mediated these symptoms.

The first hypothesis was not supported, as neither sexual identity, nor behaviour, significantly predicted probable psychosis after controlling for covariates. The second hypothesis was partially supported, as sexual identity and behaviour significantly predicted increased paranoia after controlling for co‐occurrence of AVH, while sexual identity did not significantly predict AVH after controlling for co‐occurrence of paranoia. However, sexual behaviour remained a significant predictor of AVH. The final hypothesis was partially supported as bullying, drug use, and social support partially mediated the relationship between sexual minority status and paranoia. Unexpectedly, discrimination (sexuality) had a negative effect. Furthermore, no significant total effects were found between sexual minority status and AVH in the mediation analysis.

The findings regarding sexual minority status and probable psychosis appear to go against the literature. The findings by Bolton and Sareen (2011) relied on the self‐report of receiving a diagnosis of psychotic disorder/episode, which is considerably less reliable than the probable psychosis variable used in the APMS 2007, while Gevonden *et al*. (2013) only assessed the presence of any psychotic symptom which is not equivalent to clinically relevant psychosis. However, using the same data set as the current study, Chakraborty *et al*. (2011) found both sexual identity and behaviour were significantly associated with probable psychosis. Upon analysis, the number of respondents classified as non‐heterosexual in terms of sexual identity and behaviour was substantially lower in the current study, though the number classified as heterosexual was equivalent. This may be due to differences in the way that groups were defined. For example, participants with missing relevant data in the current study were excluded during analyses. Proxy interviews were also included in the study by Chakraborty *et al*., which were interviews conducted with a respondent's family member, carer, or another person who knew the respondent well, in the case they were unable to undertake an interview alone due to mental or physical incapacity reasons (McManus *et al*., 2009). It also appears that they did not adjust for potential covariates. Despite these differences, the relationship between sexual identity and probable psychosis remained at trend level in the current study, though sexual behaviour did not predict probable psychosis. These findings would benefit from replication using independent samples in future studies.

The relationship between both sexual identity and behaviour with paranoia across all logistic models, as well as in terms of total effects in the mediation analysis, is in line with past research and theoretical expectations. Other minority groups such as ethnic minorities have a particularly high risk of paranoia (Janssen *et al*., 2003; Veling *et al*., 2007), and the current study shows sexual minorities share this risk. Bullying and lack of social support partially mediated this relationship, suggesting that issues around social identity and self‐esteem may play a part in the increased risk of paranoia in minority groups (McIntyre *et al*., 2016), and could relate to increased threat anticipation (Freeman *et al*., 2002). Conversely, recent discrimination based on sexuality had a negative effect in the mediation analyses assessing sexual minority status and paranoia, contradicting past findings (Gevonden *et al*., 2013). This may be due to the discrimination variable and the symptom variable both only encapsulating participant experiences within the past 12 months at the point of the interview. As a result, it may not be possible to assess cause and effect between the two. It is also possible that the negative effect is due to high covariance with other mediator variables in the model. For example, it has been found that bullying in non‐heterosexual men is often attributed to sexual orientation (King *et al*., 2011). The definition of bullying in the current study means that overlap between lifetime experience of bullying and the definition of discrimination cannot be disentangled. Finally, drug use was also found to partially mediate the relationship between both sexual identity/sexual behaviour and paranoia, supporting previous findings (Gevonden *et al*., 2013). Drug use is particularly elevated in sexual minorities (Corliss *et al*., 2011) and relates to risk of psychotic symptoms (Miller *et al*., 2001), representing another pathway to paranoia in sexual minorities.

The hypothesis that adversity can have specific effects on psychotic experiences was largely supported (Bentall *et al*., 2012). Significant effects of sexual identity on AVHs were not found after controlling for covariates and co‐occurrence of paranoia in the logistic regression model, or in the mediation analysis in terms of the total effects for both sexual identity and behaviour on AVH. Although a significant indirect effect was found between sexual identity and AVH, the lack of a significant total effect may invalidate further exploration (Baron & Kenny, 1986). Overall, the analyses using sexual identity as the independent variable support the idea that symptoms of paranoia and AVH may involve different psychological pathways and mechanisms (Bentall, 2004).

Contrary to the specificity hypothesis, sexual behaviour significantly predicted AVH before and after controlling for covariates and co‐occurrence of paranoia. It should be acknowledged that sexuality is a broad concept, with sexual identity and behaviour often being discordant concepts with varying outcomes (Igartua, Thombs, Burgos, & Montoro, 2009; Pathela *et al*., 2006). It is possible that differences will occur when focusing on a sexually active population and that there were unobserved risk factors for AVH which are particular to the sexually active non‐heterosexual group. For example, non‐heterosexuals are much more likely to have experienced sexual violence (Garofalo *et al*., 1999) and it is known that sexual abuse is a strong predictor of AVH (Bentall *et al*., 2014).

It should be noted that being of non‐white ethnicity strongly predicted increased odds of paranoia in the logistic models assessing both sexual identity and sexual behaviour which controlled for covariates. This provides further evidence that ethnic minorities have a specifically elevated risk of paranoia, as ethnicity had no significant effects in the models of sexual identity/behaviour and AVH.

A number of important limitations to the current study should be acknowledged. Primarily, there are issues arising from the cross‐sectional, correlational nature of the study. Causality cannot be directly inferred as measurement of variables are taken concurrently. The PSQ measures of psychosis symptoms also only reflect experiences that occurred within the last 12 months, and may not capture those with lifetime experiences of symptoms. However, this may mean that estimates of symptom prevalence are conservative. Similarly, measures of discrimination and drug use also related to the past 12 months, so it is difficult to assess the temporal sequence of these measures and symptoms. It is possible that measures of lifetime experiences may be more representative of vulnerability to psychosis and would be a more valid measure for the associated variables. It is also possible some potential confounding variables have not been controlled for. For example, urbanicity is linked to higher incidence of psychosis (Heinz *et al*., 2013) and it is known that proportions of non‐heterosexual groups are higher in cities (Ghaziani, 2019). Unfortunately, such information was not available within the current data set. Finally, the study reflects the experience of psychosis symptoms within the general population, and it is possible that a clinical sample may show different effects.

This study is the first to examine the effects of sexual minority status on specific symptoms of psychosis, as opposed to broader concepts of diagnosis or symptoms in general. Therefore, the results highlight possible mechanisms by which sexual minority status can lead to paranoia could be examined. The mediators in the study likely represent only a few of the disadvantages that sexual minorities face, but are perhaps a starting point for further exploration. The current study may have a number of implications. It has been shown that sexual minorities whom already face social disadvantage are more likely to develop symptoms of paranoia, and AVH to a lesser extent. This may result in even further disadvantage for sexual minorities on the basis of having worse mental health outcomes. Wider factors that lead to sexual minorities facing increased bullying, drug use, and reduced social support, which likely relate to a wider picture of prejudice and unacceptance of non‐heterosexuality in society, also need to be addressed.

Future research should attempt to further explore the psychological mechanisms relating to paranoia amongst sexual and ethnic minorities. Using direct measures of self‐esteem or social identity could provide further evidence about the pathways from social adversity to paranoia symptoms. Research regarding the complex nature of sexuality should also be a focus and would allow a deeper understanding of the ways sexuality affects psychological experience.
